# An Experimental and Clinical Research into Certain Problems Relating to Surgical Operations

**Published:** 1902-09

**Authors:** 


**An Experimental and Clinical Research into certain Problems relating to Surgical Operations.** By GEORGE W. CRILE, M.D. Pp. 200. Philadelphia: J. B. Lippincott Company. 1901.

The problems relating to surgical operations dealt with in this book are very important ones, and the investigations carried on by the author have thrown much light on them. All Dr. Crile's statements are substantiated by facts, and are therefore of great value to the surgeon, and the perusal of the book will give valuable hints for operative work. With regard to the question—which is of so much interest at the present time—as to the value and safety of anæsthesia produced by injection of a solution of cocaine into the subarachnoid space, several very important facts are given. The effect of cocaine when injected into a nerve is fully considered, and suggestions for its use in surgery are made, illustrated by cases in which this has been done with success. In operating for large tumours or infiltrating malignant growths of the neck, it is sometimes an important matter to decide whether temporary closure of the carotid artery can be safely made, and whether, if the vagus nerve is involved in the growth, a portion of it can be safely excised. These are problems on which the author gives us valuable information. We very strongly commend the scientific character of the work and the clear style of the author.

